# Phase Diagrams of Ternary π-Conjugated Polymer Solutions for Organic Photovoltaics

**DOI:** 10.3390/polym13060983

**Published:** 2021-03-23

**Authors:** Jung Yong Kim

**Affiliations:** School of Chemical Engineering and Materials Science and Engineering, Jimma Institute of Technology, Jimma University, Post Office Box 378 Jimma, Ethiopia; jungyong.kim@ju.edu.et

**Keywords:** conjugated polymer, phase diagram, ternary, polymer solutions, polymer blends, Flory-Huggins theory, polymer solar cells, organic photovoltaics, organic electronics

## Abstract

Phase diagrams of ternary conjugated polymer solutions were constructed based on Flory-Huggins lattice theory with a constant interaction parameter. For this purpose, the poly(3-hexylthiophene-2,5-diyl) (P3HT) solution as a model system was investigated as a function of temperature, molecular weight (or chain length), solvent species, processing additives, and electron-accepting small molecules. Then, other high-performance conjugated polymers such as PTB7 and PffBT4T-2OD were also studied in the same vein of demixing processes. Herein, the liquid-liquid phase transition is processed through the nucleation and growth of the metastable phase or the spontaneous spinodal decomposition of the unstable phase. Resultantly, the versatile binodal, spinodal, tie line, and critical point were calculated depending on the Flory-Huggins interaction parameter as well as the relative molar volume of each component. These findings may pave the way to rationally understand the phase behavior of solvent-polymer-fullerene (or nonfullerene) systems at the interface of organic photovoltaics and molecular thermodynamics.

## 1. Introduction

Since Flory-Huggins lattice theory was conceived in 1942, it has been widely used because of its capability of capturing the phase behavior of polymer solutions and blends [1,2,3]. Specifically, in 1949, Scott and Tompa applied the Flory-Huggins model to ternary systems, such as solvent-polymer-polymer and nonsolvent-solvent-polymer [4,5,6]. Since then, Loeb and Sourirajan invented the integrally skinned asymmetric membrane in 1963 [7], so the Flory-Huggins theory has been more utilized to describe the film-formation process and morphology through nonsolvent induced phase inversion (NIPI) or immersion precipitation from the ternary nonsolvent-solvent-polymer system [8,9]. Meanwhile, the original Flory-Huggins theory has been further extended by considering polymer-size (or polydispersity) and polymer-composition dependent interaction parameters [10,11,12,13,14]. However, although this generalization of the Flory-Huggins theory contributed to the enhancement of accuracy in describing experimental data, the theory should be maintained in its simplicity, allowing the original model to still function in the scientific society [15,16,17,18,19].

Importantly, in 1976, the new π-bonded macromolecules showing the full range from insulator to metal through doping were discovered by Heeger, MacDiarmid and Shirakawa [20,21,22]. Then in 1992, the photoinduced electron transfer between a conjugated polymer and fullerene was demonstrated on a picosecond time scale [23], paving the way for the development of bulk-heterojunction (BHJ) polymer/fullerene solar cells [24,25]. Here, for the desired BHJ structural morphologies, the phase scale of the active layer should be controlled within exciton diffusion length ~10 nm in organic semiconductors [26,27] (or ~20–47 nm for nonfullerene acceptors (NFAs) [28]; ~70 nm for ordered conjugated polymers [29]; >200 nm for conjugated block-copolymer nanofibers) [30], motivating to understand the phase-separation mechanism through the calculation of ternary phase diagrams containing the binodal, spinodal, tie line, and critical point [31,32,33]. Then, the binodal curves were calculated for the ternary polyfluorene derivative-fullerene-solvent systems [34]. In the following year 2008, more progress was made in polymer thermodynamics, that is, the construction of the phase diagrams for the binary poly(3-hexylthiophene-2,5-diyl) (P3HT)/[6,6]-phenyl C_61_-butyric acid methyl ester (PC_61_BM) system based on thermal and optical analyses [35,36]. Subsequently, other binary and ternary phase diagrams have been further constructed for organic photovoltaics (OPV) [37,38,39,40,41,42,43,44,45,46,47,48,49,50,51,52,53]. Importantly, the theoretical description for the ternary solvent-polymer-fullerene and solvent-polymer-NFA systems helps to rationally understand the morphology-generation mechanism for the active layer of solar cells, although it is not as sophisticated, compared to the phase inversion membrane field [46,47,48,49,50,51,52,53].

To understand the bicontinuous morphologies in the demixed donor/acceptor blends for OPV, both thermodynamics and kinetics are usually required, concerning both the equilibrium and dynamics of liquid-liquid (L-L) and liquid-solid (L-S) phase transition [30,31,32,45,54,55]. Furthermore, L-L demixing could be subdivided into spinodal decomposition (SD) and nucleation and growth (NG), whereas the L-S demixing could be crystallization, gelation, and vitrification depending on the properties of materials [55,56]. To date, regarding the L-L and L-S demixing of ternary conjugated polymer solutions, there have been two kinds of viewpoints in literature [48,57,58,59]. One is the 1–2 nm thick surface-directed SD followed by crystallization in the P3HT/PC_61_BM system [57], and the other is its reverse process, i.e., the initial crystallization (self-assembly) of P3HT followed by the lateral/vertical diffusion of PC_61_BM molecules leading to the NG process [59]. However, it is expected that the sequence of the above L-L to L-S (or its reverse) phase transition might be dependent on the time allowed for crystallization [60] through energy minimization and packing from the ternary polymer solution during a non-equilibrium spin-casting process.

In previous studies [18,19], the phase behavior of a binary P3HT/PC_61_BM blend was elucidated, leading to temperature-composition phase diagrams [18]. Then the binary low bandgap conjugated polymer solutions and blends were investigated, resulting in various phase diagrams as a function of solvent species, polymer, and chain length [19]. In this study, our interests were further extended to ternary polymer solutions such as solvent-polymer-fullerene and solvent-polymer-NFA systems, in which the composition effect on the phase behavior was examined through ternary phase diagrams. Note that, for this work, two assumptions were made as follows: (1) A homogenous ternary solution is prepared in the first stage. (2) Then through the quenching process, the L-L demixing will precede any crystallization. Then, based on these assumptions, a semicrystalline polymer can be treated as an amorphous chain molecule [61], which could be phase-separated through the L-L phase transition [3,16]. For example, the ternary P3HT solutions were studied as a function of temperature, molecular weight, solvent species [CB, chloroform (CF), toluene (TOL)], processing additive (DIO, and ODT), and electron acceptor (PC_61_BM, PC_71_BM, and ITIC). Here, DIO and ODT stand for 1,8-diiodooctane and 1,8-octanedithiol, respectively, whereas ITIC denotes 3,9-bis(2-methylene- (3-(1,1-dicyanomethylene)-indanone))-5,5,11,11-tetrakis(4-hexyl phenyl)-dithieno[2,3-d: 2’,3’-d’]-s-indaceno[1,2-b:5,6b’] dithiophene [62]. Then, our analysis was extended to other high-performance low bandgap polymers such as PTB7 and PffBT4T-2OD. Here, PTB7 denotes poly[[4,8-bis[(2-ethylhexyl)oxy]benzo[1,2-b:4,5-b’]dithiophene-2,6-diyl] [3- fluoro-2-[(2-ethylhexyl)carbonyl]thieno[3,4-b]thiophenediyl]] [63,64,65], and PffBT4T-2OD stands for poly[(5,6-difluoro-2,1,3-benzothiadiazol-4,7-diyl)-alt-(3,3‴-di (2-octyldodecyl) 2,2′;5′,2″; 5″,2‴-quarterthiophen-5,5‴-diyl)] [44,66]. Finally, the phase diagrams of ternary CB/PTB7/ITIC and CB/PffBT4T-2OD/ITIC systems were constructed. Note that, in this work, all the binodal, spinodal, tie line, and critical point data were calculated based on the Flory-Huggins theory with a constant interaction parameter and the molar volume ratio of each component.

## 2. Experimental Methods

Regioregular P3HT [*M_n_* = 22.0 kg/mol, *M_w_* = 46.2 kg/mol, polydispersity index (PDI) = 2.1, and molecular formula = (C_10_H_14_S)_n_] was purchased from Rieke Metals. PC_61_BM and PC_71_BM were provided from Nano-C. The molecular weight of P3HT was measured by a gel permeation chromatograph (GPC) (PL-GPC50) equipped with a refractive index detector using THF as an eluent. The columns were calibrated using a standard polystyrene sample. Contact angles of water were measured for the P3HT:PC_61_BM (=1:0.8 and 1:1 weight ratio) blend films on a glass slide using a contact angle analyzer (Phoenix 300+/LCA10) as explained in previous studies [18,19].

## 3. Theoretical Methods

The Flory-Huggins lattice model [1,2,3,14,67] was employed to construct the ternary phase diagrams. Here, the Gibbs free energy of mixing ($\Delta G_{mix}$) for a ternary system is given as follows [3,4,5,6,10,11,15]:

(1) $$\frac{\Delta G_{mix}}{RT}=n_{1}\ln\phi_{1}+n_{2}\ln\phi_{2}+n_{3}\ln\phi_{3}+\chi_{12}n_{1}\phi_{2}+\chi_{13}n_{1}\phi_{3}+\chi_{23}n_{2}\phi_{3}$$

where *R* is the Gas constant, *T* is temperature (K), $n_{i}$ is the number of moles of component *i*, $\phi_{i}$ is the volume fraction of component *i*, and $\chi_{ij}$ is Flory-Huggins interaction parameter between components *i* and *j*. In Equation (1), it is notable that $\chi_{ternary}=\chi_{123}$ (a ternary interaction parameter) is assumed to be zero. Furthermore, in this study, the $\chi_{ij}$ parameter is defined as follows [46,47,48,49,50,51,52,53,67]:

(2) $$\chi_{ij}=\frac{v_{1}}{RT}{\left(\delta_{i}-\delta_{j}\right)}^{2}+0.34$$

where $\nu_{1}$ is molar volume of component 1 (usually, solvent), and $\delta_{i\;\mathrm{or}\;j}$(= $\sqrt{\delta_{d}^{2}+\delta_{p}^{2}+\delta_{h}^{2}}$ where $\delta_{d}$, $\delta_{p}$, and $\delta_{h}$ are the physical quantities from dispersion force, polar force, and hydrogen bonding, respectively) is the solubility parameter of component *i* or *j*, estimated from the relationship of $\delta_{i}\propto \sqrt{\gamma_{sv}}$ [18,19,34,46]. Here, the surface energy ($\gamma_{sv}$) for solid-vapor could be numerically calculated from the contact angle (θ) data according to Li and Neumann [68,69]:

(3) $$\cos\theta =-1+2\sqrt{\frac{\gamma_{sv}}{\gamma_{lv}}}e^{-\beta {\left(\gamma_{lv}-\gamma_{sv}\right)}^{2}}$$

where β = 0.0001115 (m^2^/mJ)^2^, and $\gamma_{lv}$ is surface energy for liquid-vapor. Importantly, in many polymer systems, $\chi_{ij}$ was reported to be a composition-dependent parameter [10,11,12,14]. However, in this study $\chi_{ij}$ is assumed to be a constant because, to date, there has been no available data for this composition dependence in conjugated polymer science.

The chemical potential ($\Delta \mu_{i}$) of component *i*, i.e., the first derivative of the free energy, could be calculated using the equations below [3,4,5,6,10,11,15]:

(4) $$\frac{\Delta \mu_{1}}{RT}=\ln\phi_{1}+\left(1-\phi_{1}\right)-s\phi_{2}-r\phi_{3}+\left(\chi_{12}\phi_{2}+\chi_{13}\phi_{3}\right)\left(\phi_{2}+\phi_{3}\right)-\chi_{23}s\phi_{2}\phi_{3}$$

(5) $$\frac{\Delta \mu_{2}}{RT}=\ln\phi_{2}+1-\left(\frac{1}{s}\phi_{1}+\phi_{2}+\frac{r}{s}\phi_{3}\right)+\left(\chi_{12}\frac{1}{s}\phi_{1}+\chi_{23}\phi_{3}\right)\left(\phi_{1}+\phi_{3}\right)-\chi_{13}\frac{1}{s}\phi_{1}\phi_{3}$$

(6) $$\frac{\Delta \mu_{3}}{RT}=\ln\phi_{3}+1-\left(\frac{1}{r}\phi_{1}+\frac{s}{r}\phi_{2}+\phi_{3}\right)+\left(\chi_{13}\frac{1}{r}\phi_{1}+\chi_{23}\frac{s}{r}\phi_{2}\right)\left(\phi_{1}+\phi_{2}\right)-\chi_{12}\frac{1}{r}\phi_{1}\phi_{2}$$

where $s=\nu_{1}/\nu_{2}$, $r=\nu_{1}/\nu_{3}$, and $s/r=\nu_{3}/\nu_{2}$. Here, $\nu_{2}$ and $\nu_{3}$ are the molar volumes of components 2 and 3, respectively. The binodal curve, also called the miscibility gap, could be calculated based on the below equilibrium condition [1,2,3,14]:

(7) $$\Delta \mu_{1}^{\alpha }=\Delta \mu_{1}^{\beta }\;\;\;\left(i=1,\;2,\;3\right)$$

where α and β indicate two different phases, i.e., a polymer lean phase and a polymer rich phase. In the case of the spinodal curve, i.e., the second derivative of the free energy, it could be calculated from the equation below [3,4,5,6,10,11],

(8) $$\left|G\right|=\left|\begin{array}{cc} G_{22} & G_{23}\\ G_{32} & G_{33} \end{array}\right|=0$$

where $G_{ij}=\left(\partial^{2}\Delta {\bar{G}}_{mix}/\partial \phi_{i}\partial \phi_{j}\right)v_{ref}$. Here $\Delta {\bar{G}}_{mix}$ is Gibbs free energy of mixing with unit volume basis and $\nu_{ref}$ is the molar volume of the reference component ($=\;v_{1}$). Then, $G_{23}G_{33}={\left(G_{23}\right)}^{2}$. Finally, the critical point for a ternary system (when $\chi_{ij}$ is a constant parameter) could be calculated based on the equation below [3,4,5,6,10,11]:

(9) $$1-s{\left(\frac{\phi_{1}^{c}}{\phi_{2}^{c}}\right)}^{2}-2\frac{G_{22}}{G_{23}}\left(1-\frac{G_{22}}{G_{23}}\right)-1-r{\left(\frac{\phi_{1}^{c}}{\phi_{3}^{c}}\right)}^{2}{\left(\frac{G_{22}}{G_{23}}\right)}^{3}=0$$

where $\phi_{1}^{c}$, $\phi_{2}^{c}$, and $\phi_{3}^{c}$ are the volume fractions of component 1, 2, and 3 at critical point, respectively. However, if $\chi_{ij}$ were a function of composition [10,11,12], the aforementioned formula should be additionally modified, e.g., Equation (9) should be expanded to $G_{222}G_{33}^{2}-3G_{223}G_{23}G_{33}+G_{233}G_{23}^{2}-G_{22}G_{23}G_{333}=0$ with $G_{222}=\partial G_{22}/\partial \phi_{2}$, $G_{223}=\partial G_{22}/\partial \phi_{3}$, $G_{233}=\partial G_{23}/\partial \phi_{3}$, and $G_{333}=\partial G_{33}/\partial \phi_{3}$ [5,6,10,11]. Finally, the results from the aforementioned equations allow the calculation of the ternary phase diagrams containing the binodal, spinodal, tie line, and critical point if the five parameters ($\chi_{12}$, $\chi_{13}$, $\chi_{23}$, s, and r) were specified as mentioned elsewhere [10,11,15]. Note that in order to avoid trivial solutions, initial guesses for the phase composition should be close to the correct values [11], indicating a trial-and-error method is required for constructing phase diagrams.

## 4. Results and Discussion

**Figure 1:**
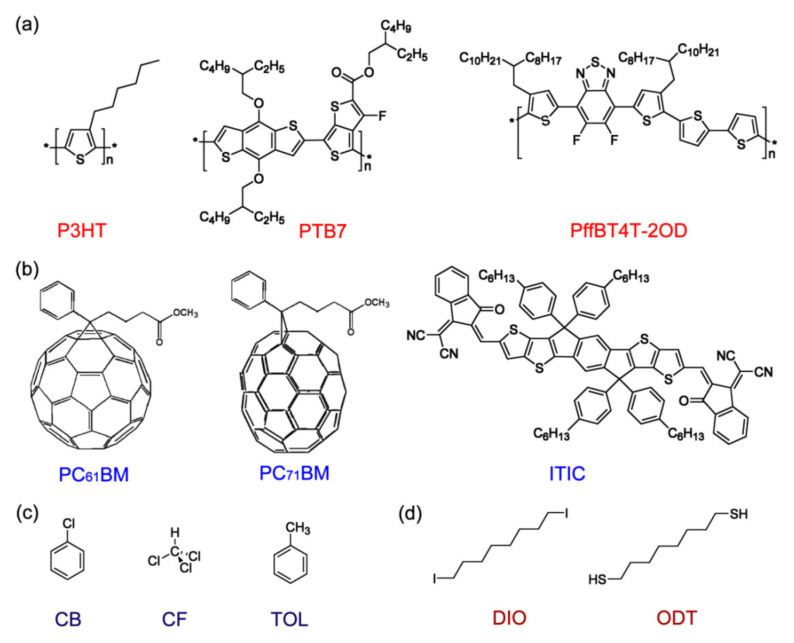
Chemical structures of (**a**) electron-donating conjugated polymers, (**b**) electron-accepting small molecules, (**c**) solvents, and (**d**) processing additives.

Figure 1 shows the chemical structures of (a) electron-donating conjugated polymers (P3HT, PTB7, and PffBT4T-2OD), (b) electron-accepting small molecules (PC_61_BM, PC_71_BM, and ITIC), (c) solvents (CB, CF, and TOL), and (d) processing additives (DIO and ODT). Table 1 and Table 2 display the characteristic properties of polymers, electron acceptors, solvents, and additives, from which the five parameters ($\chi_{12}$,$\chi_{13}$,$\chi_{23}$,s and r) were estimated (see Table 3). Note that in this study, the polymer was assumed to be monodisperse, indicating that PDI was not taken into account.

**Figure 2:**
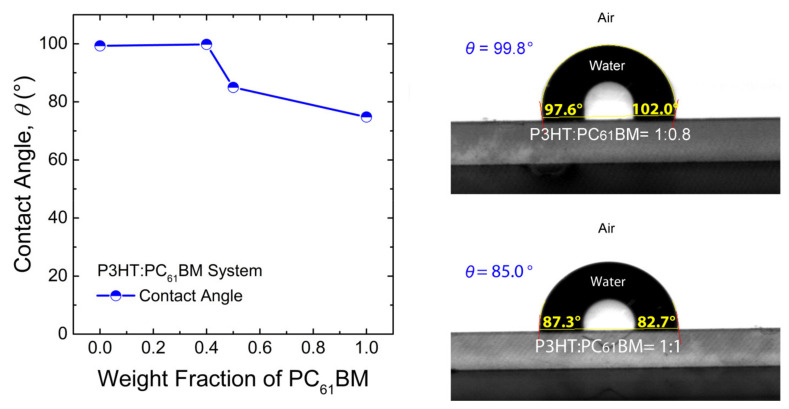
Composition dependence of the water contact angle for the P3HT:PC_61_BM system. Contact angle data for pure P3HT and PC_61_BM could be found in our previous studies [18,19]. Note that the reported contact angle is the average value of the left and right angles.

Here, $\chi_{ij}$ was estimated from the solubility parameter (*δ*), obtained from contact angle (*θ*) measurements and literature sources [18,19,44,53,65,70,71,72,73,74]. However, if *θ* is measured for the polymer/fullerene blends, it will not provide any decoupled surface energy ($\gamma_{sv}$) for each component. Hence, the composition-dependent interaction parameter is not available through the methodology of contact angle measurement. However, for characterization purposes, the contact angles for the blend samples, P3HT:PC_61_BM = 1:0.8 and 1:1, were measured. As shown in Figure 2, the data was not linearly proportional to the blend ratio, indicating that other factors (e.g., PC_61_BM miscibility/solubility limit) [35] might also be involved in the determination of surface properties. Therefore, in this work, only the contact angle data from the pure materials were considered when estimating the $\chi_{ij}$ parameters.

**Figure 3:**
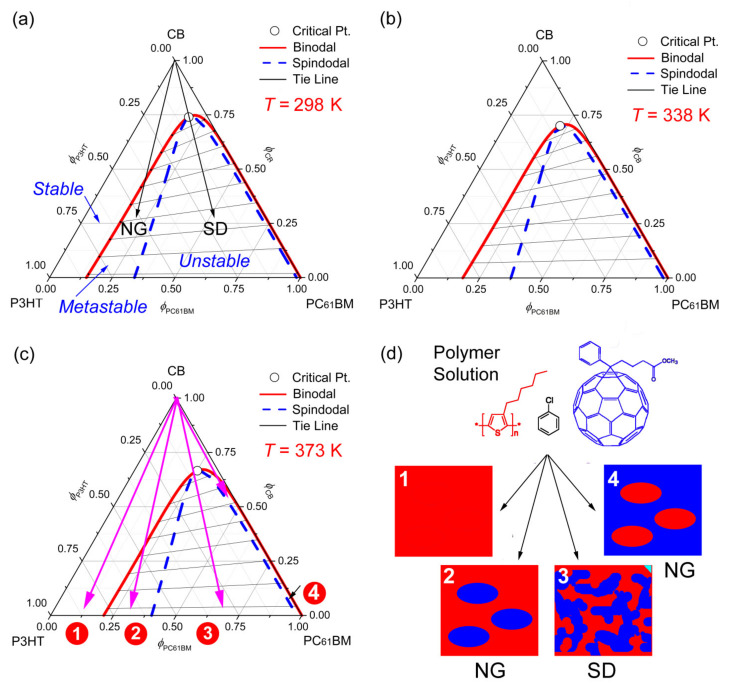
Phase diagrams for the ternary CB/P3HT/PC_61_BM system as a function of temperature. (**a**) *T* = 298 K: $\chi_{12}=0.45$, $\chi_{13}=0.90$, and $\chi_{23}=1.50$. (**b**) *T* = 338 K: $\chi_{12}=0.44$, $\chi_{13}=0.83$, and $\chi_{23}=1.36$. (**c**) *T* = 373 K: $\chi_{12}=0.43$, $\chi_{13}=0.78$, and $\chi_{23}=1.27$. Here, for P3HT, *M_n_* = 22 kg/mol, *s* = 0.005071 and *r* = 0.167068. (**d**) A schematic explanation for a film-forming process through the four cases: (1) Single homogeneous phase. (2) Nucleation and growth of the polymer-rich phase. (3) Spinodal decomposition. (4) Nucleation and growth of the polymer-lean phase.

Figure 3 shows the phase diagrams of the ternary CB/P3HT/PC_61_BM system at three different temperatures, (a) 298 K, (b) 338 K, and (c) 373 K. Among these, Figure 3a displays two representative mechanisms for the L-L demixing processes, which are the nucleation and growth (NG) and the spinodal decomposition (SD) [54,55]. Importantly, as shown in Figure 3, the metastable and unstable regions (i.e., the miscibility gap) defined by the binodal and spinodal curves are diminished with increasing temperature. For example, the critical points were downshifted from the top vertex (CB) by exhibiting ($\phi_{1}^{c}$, $\phi_{2}^{c}$, $\phi_{3}^{c}$) = (0.74, 0.07, 0.19) at 298 K, (0.70, 0.08, 0.22) at 338 K, and (0.67, 0.08, 0.25) at 373 K. Furthermore, if a linear relationship between $\phi_{2}^{c}$ and *T* were assumed, the equation ($\phi_{2}^{c}$ = 1.51972 × 10^−4^ · *T* + 0.02682) would be obtained through the linear fit (see Figure S1 in the Supplementary Materials (SM)). Hence, the phase behavior as a function of temperature indicates that the ternary system may show an upper critical solution temperature (UCST) phase behavior, as expected from most polymer solutions without any specific interaction such as hydrogen bonding. Figure 3d shows a schematic explanation for the film-forming process from a ternary polymer solution according to the four cases displayed in Figure 3c. The first case indicates a homogenous P3HT/PC_61_BM phase, in which PC_61_BM molecules may be dissolved in the amorphous region of P3HT (i.e., forming a solid solution). The second describes an NG process in the P3HT-rich phase. The third displays a SD process. Finally, the fourth is an NG process again in the P3HT-lean phase although it is rarely probable due to a limited area in the diagram. Importantly, it is notable that, for OPV applications, most polymer/fullerene systems have the composition in the range of ‘polymer: fullerene = 1:0.8 to 1:4′ (hence, it may be included in Case 3). However, it is noteworthy that, although some amorphous polymer/fullerene systems [34,46] were reported to contain circular domain structures in a film, if the OPV devices displayed that morphology, it indicates that those circular domains (e.g., PC_61_BM aggregation) might be electrically/physically interconnected within the charge hopping range (just like Case 3, allowing ambipolar transport in polymer/fullerene blend films). Furthermore, interestingly, Kim and Frisbie reported a metastable region (~30–50% PC_61_BM) in the temperature-composition phase diagram for the binary P3HT/PC_61_BM system [35]. Here, the phase diagram of the ternary CB/P3HT/PC_61_BM system reminisces the experimental results of Kim and Frisbie when $\phi_{solvent}=0$.

**Figure 4:**
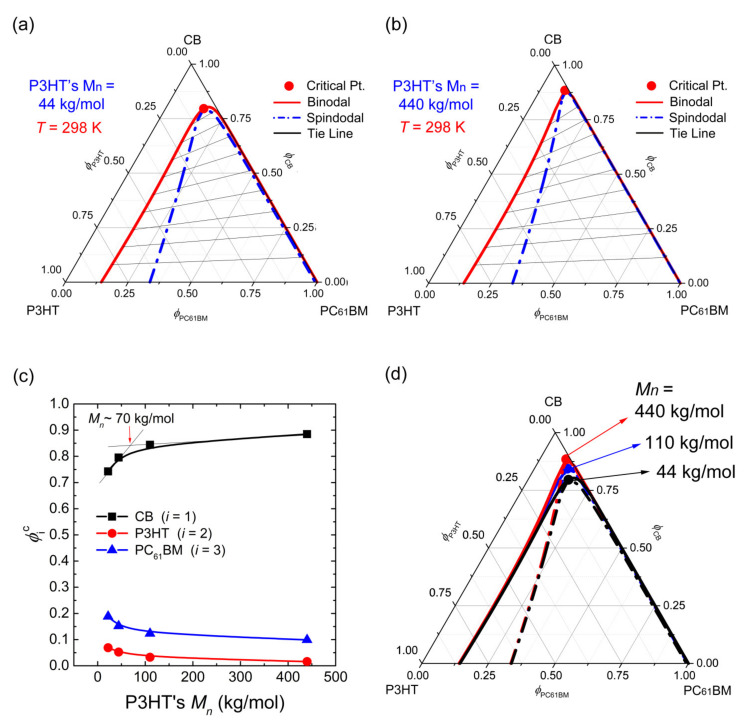
Phase diagrams for the ternary CB/P3HT/PC_61_BM system as a function of the molecular weight of P3HT, when $\chi_{12}=0.45$, $\chi_{13}=0.90$, and $\chi_{23}=1.50$ at constant T = 298 K: (**a**) P3HT’s M_n_ = 44 kg/mol, s = 0.002535, and r = 0.167068, and (**b**) P3HT’s M_n_ = 440 kg/mol, s = 0.000254, and r = 0.167068. (**c**) Critical point ($\phi_{1}^{c}$, $\phi_{2}^{c}$, $\phi_{3}^{c}$ ) of the ternary CB/P3HT/PC_61_BM system as a function of molecular weight. (**d**) Comparison of the three phase diagrams with different Mn. Note that, when P3HT has M_n_ = 110 kg/mol, the physical quantities (s = 0.001014 and r = 0.167068) are used for calculating the ternary phase diagram.

Figure 4 shows the ternary phase diagrams when the *M_n_* of P3HT was increased from 22 kg/mol (recall, Figure 3a) to 44 kg/mol (Figure 4a) and then to 440 kg/mol (Figure 4b), which explains the chain length effect on the phase behavior at 298 K. As shown in Figure 4c, when *M_n_* was increased to more than 70 kg/mol, the critical point does not change significantly, indicating that the miscibility gap is less sensitive to the increase of *M_n_*. Furthermore, as shown in Figure 4d, although the critical point is moved up with increasing *M_n_*, both the binodal and spinodal curves almost overlap (Figure 4d). However, bear in mind that, although $\chi_{ij}$ could be a function of *M_n_*, here it was assumed to be a constant.

**Figure 5:**
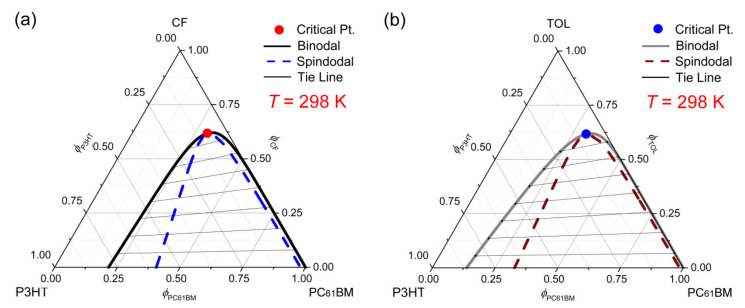
Phase diagrams for the ternary system at constant T = 298 K. (**a**) CF/P3HT/PC_61_BM: $\chi_{12}=0.37$, $\chi_{13}=0.95$, $\chi_{23}=0.92$, s = 0.004006 and r = 0.131993. (**b**) TOL/P3HT/PC_61_BM: $\chi_{12}=0.35$, $\chi_{13}=1.37$, $\chi_{23}=1.55$, s = 0.005296 and r = 0.174481. Here, CF and TOL represent chloroform and toluene, respectively.

Figure 5 shows the phase diagrams of the ternary (a) CF/P3HT/PC_61_BM and (b) TOL/P3HT/PC_61_BM systems, describing the solvent effect on the phase behavior. Here the solubility parameters of solvents are $\delta_{1}$ = 9.5 (CB), $\delta_{1}$ = 9.2 (CF), and $\delta_{1}$ = 8.9 (TOL). Hence, considering $\delta_{2}$ = 8.7 (P3HT) and $\delta_{3}$ = 11.3 (PC_61_BM), we may estimate roughly the miscibility of the ternary system. As shown in Figure 3 and Figure 5, the critical points are shifted from ($\phi_{1}^{c}$, $\phi_{2}^{c}$, $\phi_{3}^{c}$) = (0.74, 0.07, 0.19) at CB to (0.62, 0.08, 0.30) at CF and (0.62, 0.08, 0.30) at TOL, indicating there is no simple trend owing to the various intermolecular interactions among the three components (see also Figure S2 in the Supplementary Materials). Furthermore, if we consider the molecular affinity between two components based on Δδ, the CB/PC_61_BM couples (Δδ = 1.8) are more miscible than CF/PC_61_BM (Δδ = 2.1), whereas the CB/P3HT (Δδ = 0.8) are less miscible than CF/P3HT (Δδ = 0.5), indicating complicated interactions. However, it is notable that CB is more commonly used than the others (CF and TOL) in the OPV field because of its relatively high boiling point, 132 °C, allowing polymer molecules to be more organized if time is given for crystallization. In the case of TOL, although the critical point is placed lowest from the top vertex, the binodal point is (0.00, 0.86, 0.14) at $\delta_{1}$ = 0 suggesting that PC_61_BM will be easily phase-separated out from the P3HT matrix. Hence, it is very interesting to observe that, at the fixed $\chi_{23}$ value, the molecular miscibility/solubility of P3HT and PC_61_BM components could be variable depending on the processing solvent (e.g., CF, TOL, and CB) based on the prediction of Flory-Huggins theory for a ternary system. Furthermore, it is also noteworthy that, in a binary polymer/solvent system, if the third component, fullerene, is additionally incorporated into this solution, it could provide a phase-separation opportunity originating from the composition change, suggesting the usefulness of a ternary phase diagram.

**Figure 6:**
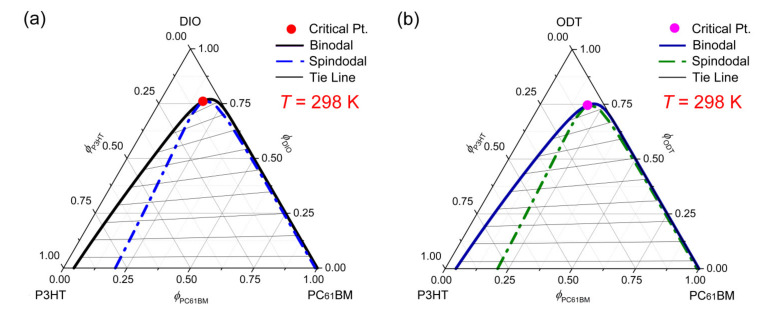
Phase diagrams of the ternary systems at constant *T* = 298 K. (**a**) DIO/P3HT/PC_61_BM: $\chi_{12}=0.42$, $\chi_{13}=1.73$, $\chi_{23}=2.47$, *s* = 0.009344 and *r* = 0.307858. (**b**) ODT/P3HT/PC_61_BM: $\chi_{12}=0.39$, $\chi_{13}=1.84$, $\chi_{23}=2.44$, *s* = 0.009196 and *r* = 0.302998. Here DIO and ODT stand for 1,8-diiodooctane and 1,8-octandithiol, respectively.

In solution-processable photovoltaic fields, additive engineering is one of the typical methods for improving the morphology of an active layer for high efficiency OPV devices [47,73,74,75,76,77]. Figure 6 shows the phase diagrams of ternary (a) DIO/P3HT/PC_61_BM and (b) ODT/P3HT/PC_61_BM systems, in which DIO ($\delta_{1}$ = 9.2) and ODT ($\delta_{1}$ = 9.1) have the boiling point of 168 °C and 270 °C, respectively. Interestingly, herein, when this additive (DIO or ODT) served as a solvent for P3HT and PC_61_BM, the Flory-Huggins theory predicted that P3HT and PC_61_BM are almost immiscible with each other (the solubility limit of PC_61_BM is only 4% in this P3HT/PC_61_BM blend film) by exhibiting the binodal point (0.00, 0.96, 0.04) at the P3HT-PC_61_BM axis (see Figure 6). This prediction suggests that the demixing process should be dependent on the choice of a solvent. However, note that this prediction was based on a specific condition, where the additive was used as a solvent for polymer and fullerene. Furthermore, another observation is that the areas defined by the spinodal curves are very wide, suggesting that it is highly probable that the polymer/fullerene blend should be phase separated in the unstable region through the spontaneous SD processes.

**Figure 7:**
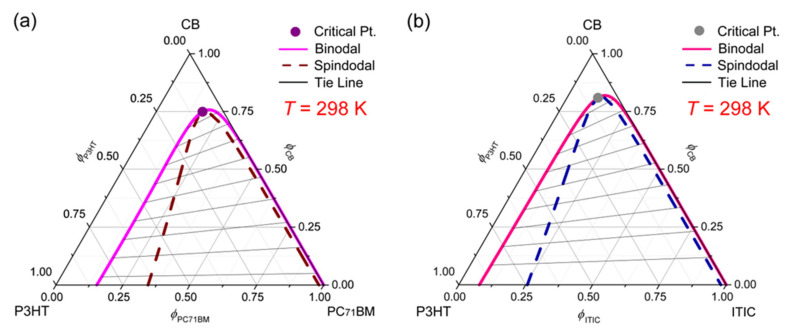
Phase diagrams for ternary systems at constant *T* = 298 K. (**a**) CB/P3HT/PC_71_BM: $\chi_{12}=0.45$, $\chi_{13}=0.84$, $\chi_{23}=1.41$, *s* = 0.005071 and *r* = 0.147613. (**b**) CB/P3HT/ITIC: $\chi_{12}=0.45$, $\chi_{13}=1.25$, $\chi_{23}=1.99$, *s* = 0.005071 and *r* = 0.071015.

Next, the electron-acceptor effect on the phase behavior was studied for the ternary systems of (a) CB/P3HT/PC_71_BM and (b) CB/P3HT/ITIC. Here, PC_71_BM has $\delta_{3}$ = 11.2 whereas ITIC has $\delta_{3}$ = 11.8, indicating that ITIC should be less miscible with P3HT ($\delta_{2}$ = 8.7) or CB ($\delta_{1}$ = 9.5) than PC_71_BM. Indeed, Figure 7 clearly exhibits that the ITIC-incorporated ternary system is less miscible than the one that incorporated PC_71_BM. However, bear in mind that the predicted results in Figure 7 are only for the case of a specific polymer, P3HT. In other words, if the polymer is replaced by another, the trend of the results will be changed accordingly, depending on the solubility parameter $\delta_{2}$.

**Figure 8:**
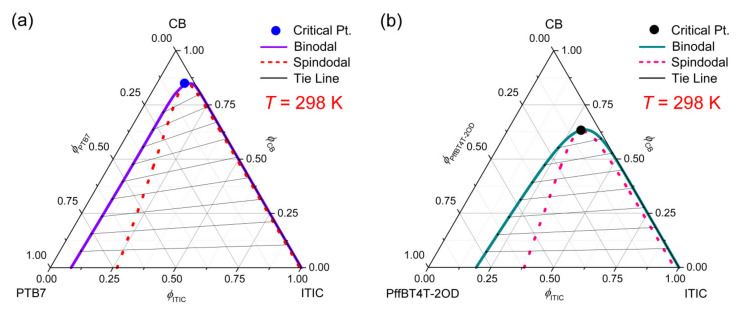
Phase diagrams for the ternary systems at constant *T* = 298 K. (**a**) CB/PTB7/ITIC: $\chi_{12}=0.42$, $\chi_{13}=1.25$, $\chi_{23}=1.88$, *s* = 0.001267 and *r* = 0.088029. (**b**) CB/PffBT4T-2OD/ITIC: $\chi_{12}=0.34$, $\chi_{13}=1.25$, $\chi_{23}=1.33$, *s* = 0.002448 and *r* = 0.088030.

Finally, the high-performance conjugated polymers such as PTB7 ($\delta_{2}$ = 8.8) and PffBT4T-2OD ($\delta_{2}$ = 9.4) were investigated by mixing one of these polymers with ITIC ($\delta_{3}$ = 11.8) and CB ($\delta_{1}$ = 9.5). Here, it is notable that PffBT4T-2OD has a very similar solubility parameter with CB, forecasting a favorable miscibility between PffBT4T-2OD and CB. Indeed, as shown in Figure 8, the miscibility gap is very small in the CB/PffBT4T-2OD/ITIC system, whereas it is very large in CB/PTB7/ITIC as expected from $\delta_{i}$ parameters.

## 5. Conclusions

The phase diagrams of ternary π-conjugated polymer solutions were constructed as a function of temperature, molecular weight, solvent species, additive, and electron acceptor. Then, our investigation was extended to the high-performance low bandgap polymers such as PTB7 and PffBT4T-2OD (PCE-11). Through this study, the results indicate: (1) The miscibility gap decreases with increasing temperature, suggesting an upper critical solution temperature (UCST) phase behavior as expected from polymer solutions without specific interactions. (2) If the *M_n_* of P3HT is increased to more than 70 kg/mol, the miscibility gap does not change much with increasing *M_n_*. (3) Among three solvents (CB, CF, TOL) tested, the chloroform displayed the smallest demixing area in the ternary phase diagram. (4) When the two additives, 1,8-diiodooctane (DIO) and 1,8-octanedithiol (ODT), were employed as a solvent in the ternary DIO(ODT)/P3HT/PC_61_BM systems, the miscibility gap was much more enlarged, indicating that these additives promoted immiscibility between P3HT and PC_61_BM. (5) If the electron-donating polymer is P3HT, the nonfullerene acceptor ITIC has a less miscibility with P3HT than does the fullerene acceptor (i.e., PC_61_BM or PC_71_BM). (6) Among the three polymers (P3HT, PTB7, and PffBT4T-2OD), the low bandgap PffBT4T-2OD polymer has the best miscibility with ITIC, demonstrating the smallest miscibility gap. Hence, our systematic study may provide a rational understanding for the demixing processes of ternary π-conjugated polymer solutions based on the cross talk between polymer photovoltaics and molecular thermodynamics. Finally, our future works may include the experimental demonstration of phase separation mechanism for the amorphous polymer/amorphous NFA, semicrystalline polymer/amorphous NFA, amorphous polymer/crystalline NFA, and semicrystalline polymer/crystalline NFA solutions from the ternary phase behavior point of view.

## Figures and Tables

**Table 1:** Solubility parameter ($\delta_{i}$ ), molecular weight (MW), molar volume ($v_{i}$ ), density (ρ ), chemical structure and reference for materials. Here, MW is *M_n_* in the case of polymers.

Materials	$\delta_{i}*$ (cal/cm^3^)^1/2^	$\delta_{i}$ MPa^1/2^	MW (g/mol)	$v_{i}$ (cm^3^/mol)	ρ (g/cm^3^)	Chemical Structure	Ref
P3HT	8.7	17.83	22,000	20,000	1.1	(C_10_H_14_S)_n_	[18,19]
PTB7	8.8	18.03	80,000	68,376	1.17	(C_41_H_53_FO_4_S_4_)_n_	[65]
PffBT4T-2OD	9.4	19.26	50,000	41,322	1.21	(C_62_H_88_F_2_N_2_S_5_)_n_	[44,70]
PC_61_BM	11.3	23.15	910	607	1.5	C_72_H_14_O_2_	[18,19]
PC_71_BM	11.2	22.95	1031	687	1.5	C_82_H_14_O_2_	[18,19]
ITIC	11.8	24.18	1428	1152	1.24	C_94_H_82_N_4_O_2_S_4_	[53]

* Note that, for calculating the $\chi_{ij}$ parameter in Equation (2), the CGS unit is used instead of the SI unit.

**Table 2:** Solubility parameter ($\delta_{i}$ ), molecular weight (MW), molar volume ($v_{i}$ ), density (ρ ), boiling point (bp), chemical structure, and reference for solvents and processing additives.

Solvent	$\delta_{i}*$ (cal/cm^3^)^1/2^	$\delta_{i}$ MPa^1/2^	MW (g/cm^3^)	$v_{i}$ (cm^3^/mol)	ρ (g/cm^3^)	bp (°C)	Chemical Structure	Ref
CB	9.5	19.47	112.56	101.41	1.11	132	C_6_H_5_Cl	[71]
CF	9.2	18.85	119.38	80.12	1.49	61	CHCl_3_	[72]
TOL	8.9	18.24	92.14	105.91	0.87	111	C_7_H_8_	[71]
DIO	9.2	18.85	336.02	186.68	1.8	168	C_8_H_16_I_2_	[73]
ODT	9.1	18.65	178.36	183.92	0.97	269	C_8_H_18_S_2_	[74]

* Note that, for calculating the $\chi_{ij}$ parameter in Equation (2), the CGS unit is used instead of the SI unit.

**Table 3:** Flory-Huggins interaction parameters and the molar volume ratios for the ternary systems.

Ternary System	Flory-Huggins Interaction Parameter *			Molar Volume Ratio *	
	$\chi_{12}$	$\chi_{13}$	$\chi_{23}$	$s=v_{1}/v_{2}$	$r=v_{1}/v_{3}$
CB/P3HT/PC_61_BM	32.7 K/*T* + 0.34	165.4 K/*T* + 0.34	345.0 K/*T* + 0.34	0.005071	0.167068
CF/P3HT/PC_61_BM	10.1 K/*T* + 0.34	177.8 K/*T* + 0.34	272.6 K/*T* + 0.34	0.004006	0.131993
TOL/P3HT/PC_61_BM	2.1 K/*T* + 0.34	307.0 K/*T* + 0.34	360.3 K/*T* + 0.34	0.005296	0.174481
DIO/P3HT/PC_61_BM	23.5 K/*T* + 0.34	414.3 K/*T* + 0.34	635.1 K/*T* + 0.34	0.009344	0.307858
ODT/P3HT/PC_61_BM	14.8 K/*T* + 0.34	448.0 K/*T* + 0.34	625.7 K/*T* + 0.34	0.009196	0.302998
CB/P3HT/PC_71_BM	32.7 K/*T* + 0.34	147.5 K/*T* + 0.34	319.0 K/*T* + 0.34	0.005071	0.147613
CB/P3HT/ITIC	32.7 K/*T* + 0.34	270.0 K/*T* + 0.34	490.5 K/*T* + 0.34	0.005071	0.071015
CB/PTB7/ITIC	25.0 K/*T* + 0.34	270.0 K/*T* + 0.34	459.3 K/*T* + 0.34	0.001483	0.088029
CB/PffBT4T-2OD/ITIC	0.5 K/*T* + 0.34	270.0 K/*T* + 0.34	294.0 K/*T* + 0.34	0.002448	0.088030

* Note that the calculation methods are included in Supplementary Materials.

## Data Availability

The data presented in this study are available on request from the corresponding author.

## Supplementary Materials

The following are available online at https://www.mdpi.com/2073-4360/13/6/983/s1, Calculation methods: the Flory-Huggins interaction parameter and the relative molar volume for the CB/P3HT/PC_61_BM system as an example. Figure S1a: Critical point ($\phi_{1}^{c}$, $\phi_{2}^{c}$, $\phi_{3}^{c}$) of the ternary CB-P3HT-PC_61_BM system as a function of temperature. (b) Linear fit for the plot of $\phi_{2}^{c}$ (P3HT) vs. temperature: $\phi_{2}^{c}$ = 1.51972 × 10 − 4·T + 0.02682. Figure S2a: Critical point ($\phi_{1}^{c}$, $\phi_{2}^{c}$, $\phi_{3}^{c}$) of the ternary Solvent/P3HT/PC_61_BM system as a function of solvent species (CB, CF, and TOL). CB, CF, and TOL stand for chlorobenzene, chloroform, and toluene, respectively. (b) The plot of $\phi_{2}^{c}\;\mathrm{vs}.\;\delta_{1}$ for clarifying the indistinguishable data shown in Figure S2a.
