# Supplementary material for: Phase Diagrams of Ternary π-Conjugated Polymer Solutions for Organic Photovoltaics
Source: Polymers (Basel). 2021 Mar 23;13(6):983. doi: 10.3390/polym13060983 (PMC8004777; doi:10.3390/polym13060983)
Supplement: Supplementary file 1 [file polymers-13-00983-s001.pdf]

Supplementary Materials

Phase Diagrams of Ternary π-Conjugated Polymer Solutions for Organic Photovoltaics

Jung Yong Kim*

School of Chemical Engineering and Materials Science and Engineering, Jimma Institute of Technology, Jimma University, Post Office Box 378, Jimma, Ethiopia

* Corresponding author

E-mail: jungyong.kim@ju.edu.et

ORCID: Jung Yong Kim: 0000-0002-7736-6858

Calculation Methods for the CB/P3HT/PC$_{61}$BM System

- **Flory-Huggins interaction parameter**: χ_{12}, χ_{13}, and χ_{23}

CB: $\delta_1 = 9.5$ (cal/cm^3)$^{1/2}$; MW_1 =112.56 g/mol; ρ_1=1.11 g/cm^3; $v_1 = \frac{MW_1}{\rho_1} = 101.41\,\mathrm{cm^3/mol}$

P3HT: $\delta_2 = 8.7$ (cal/cm^3)$^{1/2}$; $MW_2 = M_n$ =22,000 g/mol; ρ_2=1.1 g/cm^3.

PC$_{61}$BM: $\delta_3 = 11.3$ (cal/cm^3)$^{1/2}$; $MW_3 = 910$ g/mol; ρ_3=1.5 g/cm^3.

$R = 1.987\,\mathrm{cal/K\cdot mol}$

$$\chi_{12} = \frac{\frac{MW_1}{\rho_1}}{R\cdot T}(\delta_1-\delta_2)^2+0.34 = \frac{\frac{112.56\ \mathrm{g/mol}}{1.11\ \mathrm{g/cm^3}}}{1.987\,\mathrm{cal/K\cdot mol}}\frac{1}{T\,\mathrm{K}}\cdot\left(9.5\left(\mathrm{cal/cm^2}\right)^{1/2}-8.7\ \left(\mathrm{cal/cm^2}\right)^{1/2}\right)^2+0.34$$
$$= \frac{32.7\mathrm{K}}{T}+0.34$$

$$\chi_{13} == \frac{\frac{MW_1}{\rho_1}}{R\cdot T}(\delta_1-\delta_3)^2+0.34 = \frac{\frac{112.56\ \mathrm{g/mol}}{1.11\ \mathrm{g/cm^3}}}{1.987\,\mathrm{cal/K\cdot mol}}\frac{1}{T\,\mathrm{K}}\cdot\left(9.5\left(\mathrm{cal/cm^2}\right)^{1/2}-11.3\ \left(\mathrm{cal/cm^2}\right)^{1/2}\right)^2+0.34$$
$$= \frac{165.4\mathrm{K}}{T}+0.34$$

$$\chi_{23} == \frac{\frac{MW_1}{\rho_1}}{R\cdot T}(\delta_2-\delta_3)^2+0.34 = \frac{\frac{112.56\ \mathrm{g/mol}}{1.11\ \mathrm{g/cm^3}}}{1.987\,\mathrm{cal/K\cdot mol}}\frac{1}{T\,\mathrm{K}}\cdot\left(8.7\left(\mathrm{cal/cm^2}\right)^{1/2}-11.3\ \left(\mathrm{cal/cm^2}\right)^{1/2}\right)^2+0.34$$
$$= \frac{345.0\mathrm{K}}{T}+0.34$$

- **Relative molar volume: *s* and *r***

CB: $v_1 = \frac{MW_1}{\rho_1} = \frac{112.56\ \mathrm{g/mol}}{1.11\ \mathrm{g/cm^3}} = 101.41\ \mathrm{cm^3/mol}$

P3HT: $v_2 = \frac{MW_2}{\rho_2} = \frac{22,000\ \mathrm{g/mol}}{1.1\ \mathrm{g/cm^3}} = 20,000\ \mathrm{cm^3/mol}$

PC$_{61}$BM: $v_3 = \frac{MW_3}{\rho_3} = \frac{910\ \mathrm{g/mol}}{1.5\ \mathrm{g/cm^3}} = 607\ \mathrm{cm^3/mol}$

$s = \frac{v_1}{v_2} = \frac{101.41\ \mathrm{cm^3/mol}}{20,000\ \mathrm{cm^3/mol}} = 0.005071$ and $r = \frac{v_1}{v_3} = \frac{101.41\ \mathrm{cm^3/mol}}{607\ \mathrm{cm^3/mol}} = 0.167068$

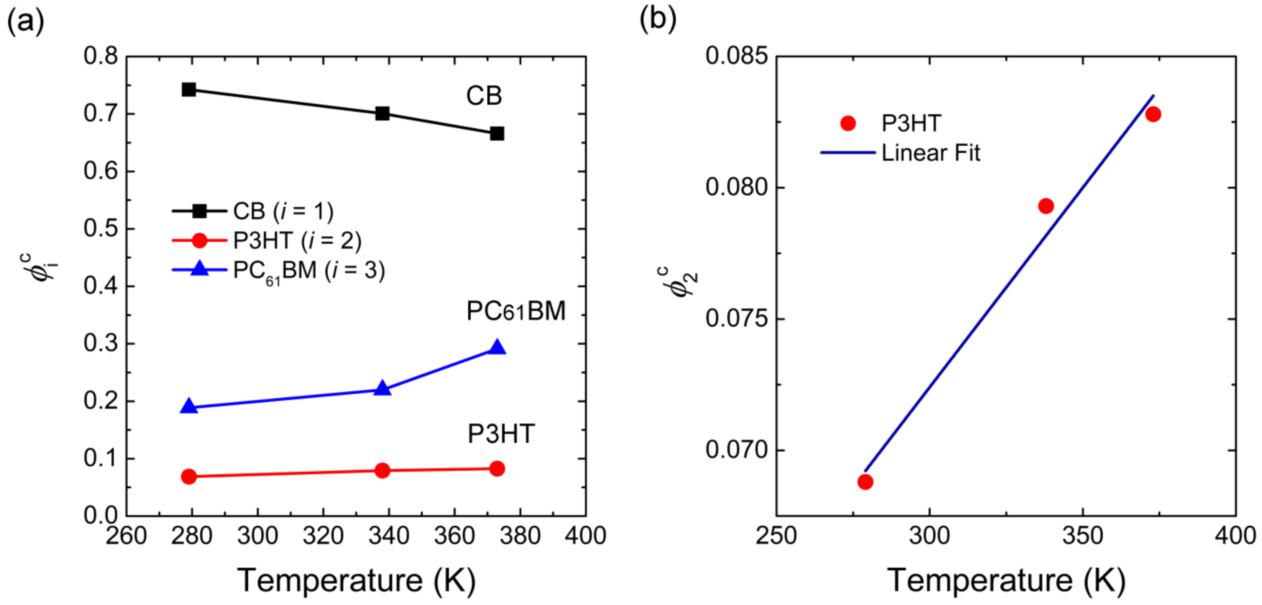

Figure S1. (a) Critical point ($\phi_1^c, \phi_2^c, \phi_3^c$) of the ternary CB-P3HT-PC_{61}BM system as a function of temperature. (b) Linear fit for the plot of ϕ_2^c (P3HT) vs. temperature: $\phi_2^c = 1.51972\times10^{-4}\cdot T + 0.02682$.

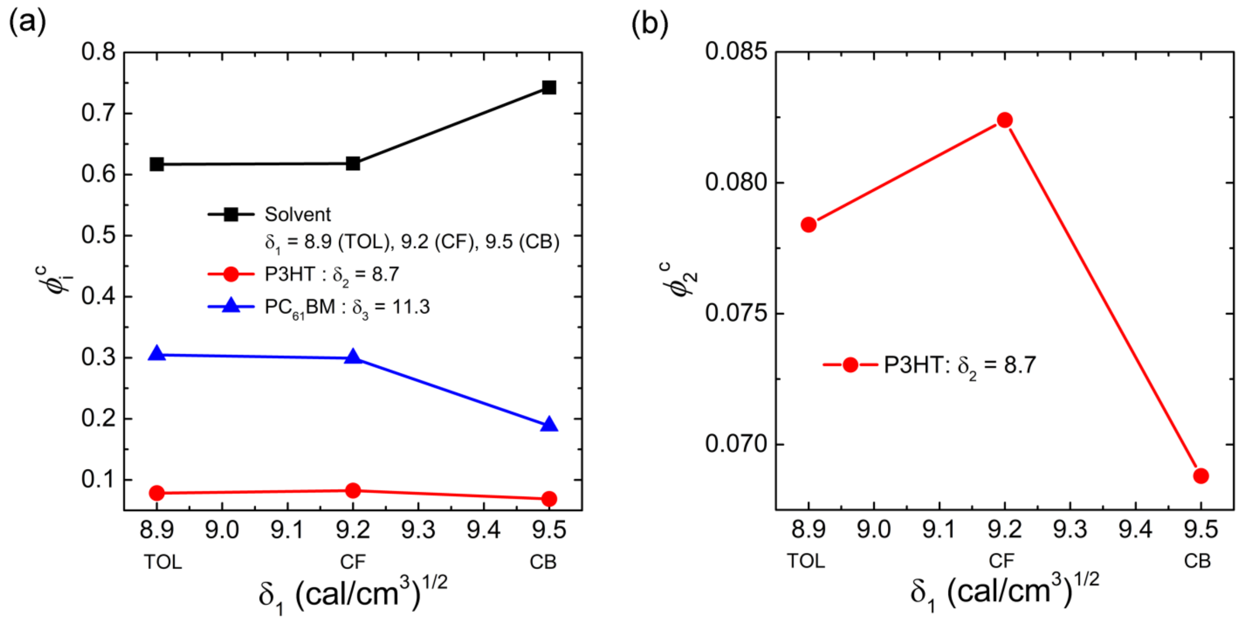

Figure S2. (a) Critical point ($\phi_1^c, \phi_2^c, \phi_3^c$) of the ternary Solvent/P3HT/$PC_{61}BM$ system as a function of solvent species (CB, CF and TOL). CB, CF, and TOL stand for chlorobenzene, chloroform, and toluene, respectively. (b) The plot of ϕ_2^c vs. δ_1 for clarifying the indistinguishable data shown in Fig S2(a).
